# Steroid Treatment in Macular Edema: A Bibliometric Study and Visualization Analysis

**DOI:** 10.3389/fphar.2022.824790

**Published:** 2022-02-22

**Authors:** Yu Lin, Xiang Ren, Danian Chen

**Affiliations:** ^1^ Research Laboratory of Ophthalmology and Vision Sciences, State Key Laboratory of Biotherapy, West China Hospital, Sichuan University, Chengdu, China; ^2^ Department of Ophthalmology, West China Hospital, Sichuan University, Chengdu, China

**Keywords:** steroids, macular edema, intravitreal injection, citespace, VOSviewer, bibliometric study

## Abstract

The use of steroids to treat macular edema (ME) is a research hotspot in ophthalmology. We utilized CiteSpace and VOSviewer software to evaluate the Web of Science Core Collection publications and to build visualizing maps to describe the research progress in this topic. There were 3,252 publications for three decades during 1988–2021. The number of studies was low during the first 14 years but has risen consistently in the following two decades. The average publications per year were only 4.8 during 1988–2002, which jumped to 113 per year during 2003–2012, and 227 per year during 2013–2021. These publications came from 83 countries/regions, with the United States, Germany, and Italy leading positions. Most studies were published in *Investigative Ophthalmology Visual Science*, and *Ophthalmology* was the most cited journal. We found 9,993 authors, with Bandello F having the most publications and Jonas JB being the most frequently co-cited. According to our research, the most popular keyword is triamcinolone acetonide (TA). Macular edema, diabetic macular edema (DME), retinal vein occlusion (RVO), dexamethasone (DEX), fluocinolone acetonide (FA), and some other keywords were commonly studied in this field. In conclusion, the bibliometric analysis provides a comprehensive overview of steroid hotspots and developmental tendencies in the macular edema study. While anti-VEGF therapy is the first-line treatment for DME and RVO-induced macular edema, steroids implant is a valid option for these DME patients not responding to anti-VEGF therapy and non-DME patients with macular edema. Combined therapy with anti-VEGF and steroid agents is vital for future research.

## 1 Introduction

The macula is responsible for central vision and locates at the center of retina, temporal to the optic nerve head (Daruich et al., 2018). Macular diseases can cause blurred vision, distorted vision, and vision loss (Chung, 2020). Macular edema (ME) develops when fluid accumulates in the macular layers due to vascular leakage, causing the macula to swell and thicken, distorting vision (Lang, 2012). It is a complication of several retinal disorders and other diseases, including diabetic retinopathy (DR), age-related macular degeneration (AMD), retinal vein occlusion (RVO), and inflammatory diseases (such as uveitis and retinal necrosis) (Welfer et al., 2011; Smith and Kaiser, 2014; Aksu-Ceylan et al., 2021; Hykin et al., 2021; Tappeiner et al., 2021). The most common causes of macular edema are DR and RVO (Daruich et al., 2018). It affects about 7 million DR patients (Yau et al., 2012), 3 million RVO patients (Rogers et al., 2010), and 40% of uveitis patients (Rothova et al., 1996; Karim et al., 2013).

Several approaches have been developed to treat macular edema and its underlying causes, such as anti-vascular endothelial growth factor (anti-VEGF) therapies, anti-inflammatory treatments, focal laser photocoagulation, carbonic anhydrase inhibitors, and vitrectomy (Mahdy et al., 2010; Supuran, 2019; Shimura et al., 2020; Aref et al., 2021; Mohan et al., 2021). Grid and focal laser photocoagulation, which was once the standard treatment for macular edema, is declining (Romero-Aroca, 2015). On the other hand, intravitreal injections have been commonly utilized to treat macular edema recently (Romero-Aroca, 2015). Anti-VEGF agents and corticosteroids are the most commonly administered drugs intravitreally (Distefano et al., 2017). Anti-VEGF agents can reduce vascular permeability and leakage (Simó et al., 2014). Anti-inflammatory steroids could help repair the blood-retinal barrier and decrease exudation (Cunningham et al., 2008; Sarao et al., 2014). Triamcinolone acetonide (TA), dexamethasone (DEX), and fluocinolone acetonide (FA) are the three most often utilized steroids in the treatment of vitreoretinal disorders (Whitcup et al., 2018). The first steroid to treat AMD and macular edema was TA, which was followed by DEX and FA (Bandello et al., 2014).

Recently sustained-release devices have been developed and approved for intravitreal steroid administration, such as an intravitreal implant that can sustain drug delivery of DEX for up to 6 months (Bucolo et al., 2018). When macular edema is caused by vitreous traction, vitrectomy can relieve the tractional forces pulling on the macula (Yoshizumi et al., 2019). Other new therapeutic agents, such as minocycline and integrin antagonists, are being examined in clinical trials to reverse or even prevent the development of macular edema (Cukras et al., 2012; Shaw et al., 2020).

Bibliometric analysis, which focuses on the literature systems and characteristics, investigates hotspots and developmental trends in the scientific literature, using qualitative and quantitative analysis to describe the relationships between citing and co-cited references and depict the contributions of various authors, countries, and journals (Gu et al., 2021; Ma et al., 2021). This approach can help develop guidelines, identify research hotspots, and predict research trends (Guler et al., 2016). As previously stated, there are many therapeutic options for treating macular edema. New progress has recently been achieved in the study of steroids, particularly in the drug delivery system of these medicines. Thus, we want to use bibliometric analysis software to map out hotspots and developmental trends in using steroids to treat macular edema over the last three decades.

## 2 Methods

### 2.1 Data Collection and Search Strategies

**FIGURE 1:**
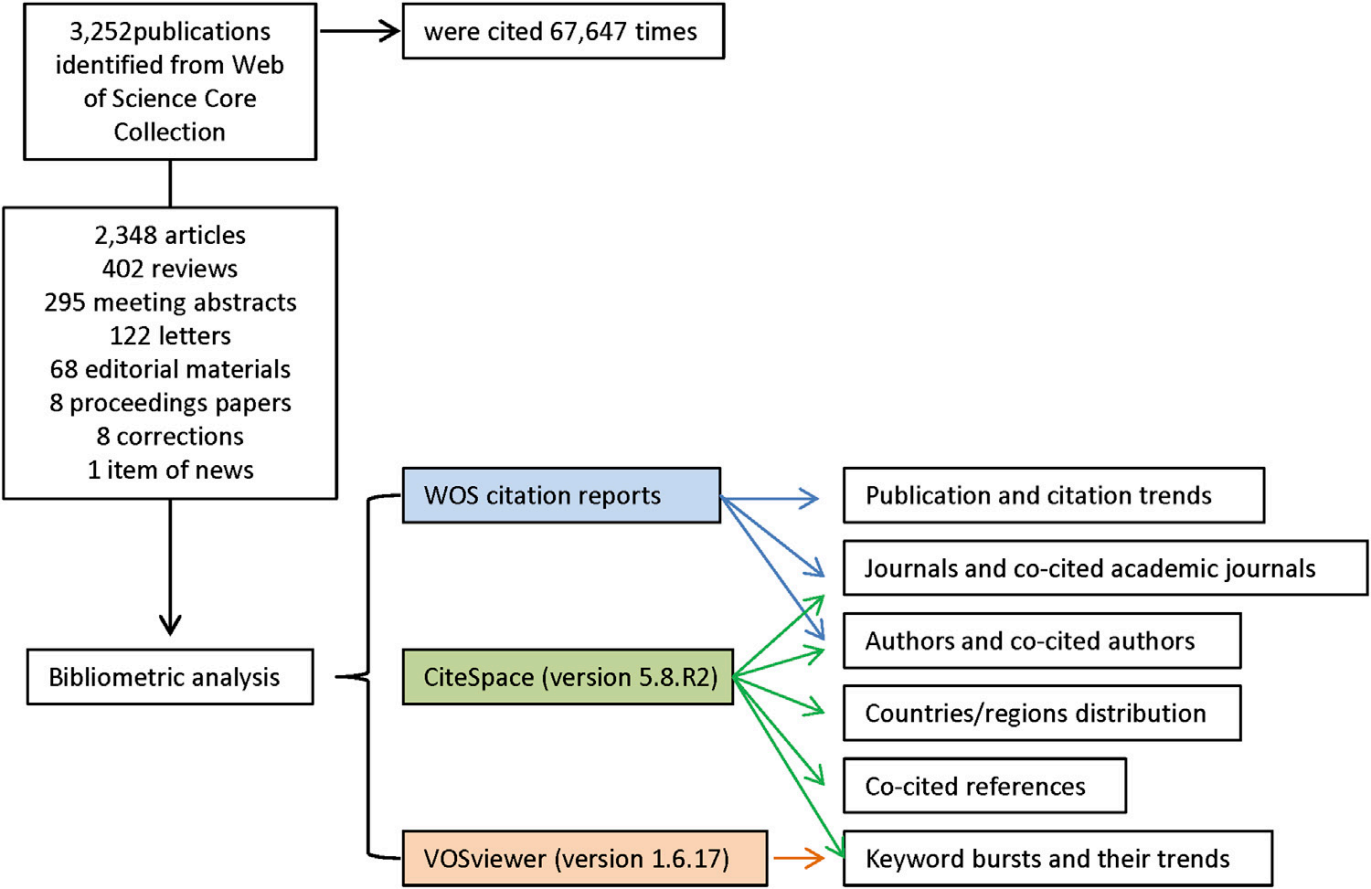
Flow diagram of the included publications and methods of bibliometric analysis.

We searched the Web of Science (WOS) Core Collection database for all literature on the treatment of macular edema with steroids. All searches were performed on a single day, 7 October 2021, to avoid biases introduced by daily database updating. The search strategies were integrated as follows: TI = (macular edema) AND TS = (glucocorticoid OR steroid OR dexamethasone OR fluortriamcinolone acetonide OR triamcinolone acetonide). A total of 3,252 records from 1988 to 2021 were identified from WOS. In the WOS citation report, these entries were cited 67,645 times in total. In the following analysis, we used all these 3,252 records, including 2,348 articles, 402 reviews, 295 meeting abstracts, 122 letters, 68 editorial materials, 8 proceedings papers, 8 corrections, and 1 item of news. The retrieved eligible publications were exported and saved as plain text files, including titles, keywords, publication dates, countries and regions, institutions, authors, publishing journals, and sums of citations (Figure 1).

### 2.2 Bibliometric Analyzing

The exported data were imported into CiteSpace version 5.8.R2 (Drexel University, Philadelphia, United States) (Chen et al., 2012; Chen, 2017) and VOSviewer version 1.6.17 (Leiden University, Leiden, Netherlands) (van Eck and Waltman, 2010) and analyzed both quantitatively and qualitatively. We used the “eliminating duplicates function” in CiteSpace to perform simple literature curation before doing the co-word analysis and document co-citation analysis. Thus the final dataset included no duplicates. The publication and citation trends were generated from the citation reports of WOS. CiteSpace was used to identify countries/regions distribution, journals and co-cited academic journals, authors and co-cited authors, keyword bursts and their trends, and co-cited references. VOSviewer was employed to map and visualize the network of hotspots (keywords) related to macular edema treated with steroids research. Hotspots were classified into disparate clusters according to co-occurrence analysis and simultaneously color-coded by time course. Detailed procedures of the enrolment and analysis are illustrated in Figure 1.

## 3 Results

### 3.1 The Publication and Citation Trends

**FIGURE 2:**
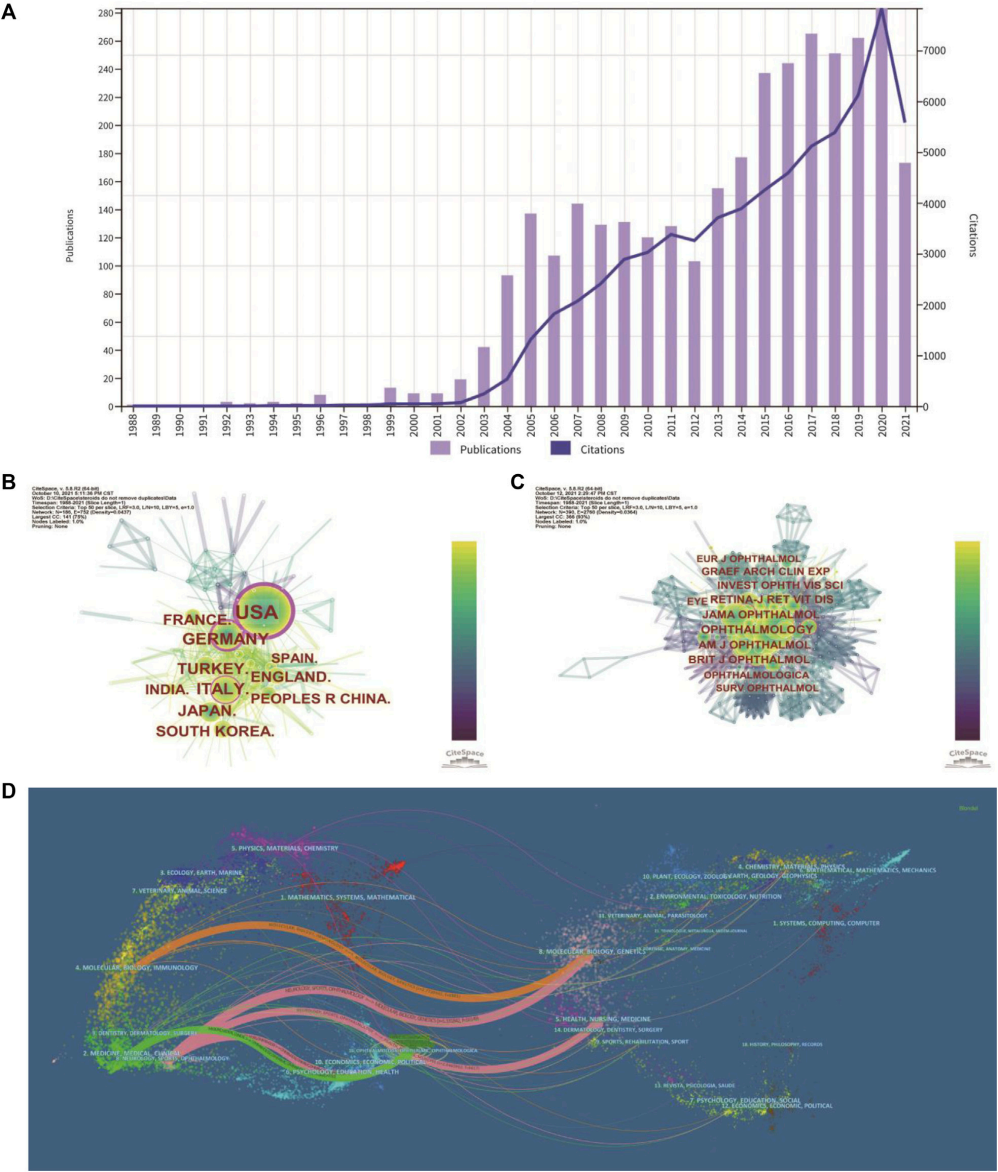
Distribution of publications and citations from different years, countries and journals. **(A)** The distribution of the bibliographic records in Set #7 **(** Table 1 **)**, showing the number of publications and citations during different years. **(B)** Distributions of publications from different countries. **(C)** CiteSpace visualization map of co-cited journals. **(D)** The dual-map overlay of journals. The dual-map shows the relationships between publications and citations, with dots representing citing journals on the left and cited journals on the right.

The number of publications and citations in a research field shows the evolution of research. The number of publications related to the treatment of macular edema with steroids by year was presented in Figure 2A. While there were less than 5 publications per year during 1988–2002, the number of publications increased to about 110 publications per year on average during 2003–2012. The number of publications increased dramatically to about 230 publications per year on average during 2013–2021 (Figure 2A). For instance, in 2020, the total number of outputs reached 283, and the number of citations increased to 7,829, which both achieved the climax (Figure 2A). In total, 3,252 publications have been cited 67,645 times, and the average number of citations per publication is 20.8 times. Likely, the citation frequency of these studies increased steadily from 1988 to 2021 (Figure 2A). These data indicated that the steroids agent is more and more commonly used to treat macular edema.

### 3.2 Analysis of Leading Countries/Region

These studies were published by researchers in 83 countries and regions worldwide. The top 10 countries/regions contributing to this research field were mainly America, Europe, and Asia (Table 1). More than one-quarter of the publications were from the United States (913, 28.066%). Germany (321, 9.868%), Italy (314, 9.653%), Turkey (242, 7.439%), and England (209, 6.425%) also made significant contributions to this topic (Table 1). The United States is the largest node on the country network map (Figure 2B). In addition, certain countries, such as the United States, Germany, and Italy, showed high centrality and were circled in the purple ring, implying that these countries may have played a crucial role in this research field (Figure 2B).

**TABLE 1:** Distribution of publications from different countries/regions.

No.	Country	Counts (%)
1	United States	913 (28.066%)
2	Germany	321 (9.868%)
3	Italy	314 (9.653%)
4	Turkey	242 (7.439%)
5	England	209 (6.425%)
6	France	204 (6.271%)
7	Japan	203 (6.240%)
8	South Korea	179 (5.503%)
9	India	153 (4.703%)
10	Peoples R China	146 (4.488%)

### 3.3 Analysis of Leading Journals and Co-Cited Journals

In total, 345 academic journals have published papers about steroids in macular edema treatment. Table 2 presented the top 8 journals contributing to this field and *Investigative Ophthalmology Visual Science (IOVS)* as the leading journal published the most papers (306, 9.407%), followed by *Retina - The Journal of Retinal and Vitreous Diseases* (247, 7.593%), *European Journal of Ophthalmology (EJO)* (146, 4.488%), and *American Journal of Ophthalmology (AJO)* (136, 4.181%). These are all major ophthalmological journals (Table 2).

**TABLE 2:** Top 8 journals and co-cited journals.

No.	Journal	Count (%)	JCR (2020)	Co-cited Journal	Citation count	JCR (2020)
1	Investigative Ophthalmology Visual Science	306 (9.407%)	Q1	Ophthalmology	2645	Q1
2	Retina - The Journal of Retinal and Vitreous Diseases	247 (7.593%)	Q1	American Journal of Ophthalmology	2327	Q1
3	European Journal of Ophthalmology	146 (4.488%)	Q3	JAMA Ophthalmology (formerly Archives of Ophthalmology)	2249	Q1
4	American Journal of Ophthalmology	136 (4.181%)	Q1	Retina - The Journal of Retinal and Vitreous Diseases	2044	Q1
5	Graefes Archive for Clinical and Experimental Ophthalmology	123 (3.781%)	Q2	British Journal of Ophthalmology	1858	Q1
6	Ophthalmology	120 (3.689%)	Q1	Investigative Ophthalmology Visual Science	1543	Q1
7	Journal of Ocular Pharmacology and Therapeutics	99 (3.043%)	Q2	Graefes Archive for Clinical and Experimental Ophthalmology	1402	Q2
8	Ophthalmologica	94 (2.890%)	Q2	Eye	1254	Q1

Q1: Quartile 1 of JCR 2020.

The impact factor (IF) represents the importance of journals in particular respective fields, with higher IF indicating publications in that journal with more frequent citations (Mueller et al., 2006). Among these 8 top journals, *Ophthalmology* has the highest IF (12.079). Co-citation analysis can also measure the degree of relationship between articles. The impact of a journal depends on its co-citation frequency. CiteSpace can show co-citations and annotate cited journals based on citation frequency (Figure 2C). Table 2 presented the top eight journals which were cited over 1,000 times, and consistent with the IF, *Ophthalmology* has been the most frequently co-cited journal (2,645 times), followed by *AJO* (2,327 times), *JAMA Ophthalmology* (previously *Archives of Ophthalmology*) (2,249 times), and *Retina - The Journal of Retinal and Vitreous Diseases* (2,044 times). According to the journal citation reports (JCR) in 2020 (Clarivate, United Kingdom), seven of the top eight co-cited journals were in the Quartile 1 (Q1), except for *Graefes Archive for Clinical and Experimental Ophthalmology* (Table 2).

CiteSpace can also show the relationships between citing journals and cited journals in the dual-map overlay of journals (Figure 2D), with dots representing citing journals on the left and cited journals on the right (Chen et al., 2014). The cited relationships are depicted by the colored lines that run from the left to the right side of the dual map. There are five main citation paths, including three pink paths, one orange path, and one green path (Figure 2D). The three pink routes show that Ophthalmology journals mostly cited Molecular or Biology or Genetics, Health or Nursing or Medicine, and Ophthalmology journals. Studies published in Molecular/Biology/Immunology journals frequently cited articles published in Molecular/Biology/Genetics journals, as indicated by the orange path. The green path indicates that research published in Medicine/Medical/Clinical journals cited papers published in Ophthalmology journals frequently.

### 3.4 Analysis of Authors and Co-Cited Authors

A total of 9,993 authors published papers related to the use of steroids to treat macular edema (Table 3). Bandello F from the Department of Ophthalmology, Vita-Salute San Raffaele University, Italy, had the highest number of published papers (77 publications, 2.367%), followed by Jonas JB (57, 1.752%), and Loewenstein A (49, 1.506%). Table 3 also shows the top 10 most frequently co-cited authors, including Jonas JB (902 times), Haller JA (597 times), and Campochiaro PA (542 times). Jonas JB from the Department of Ophthalmology, Medical Faculty Mannheim of the Ruprecht-Karls-University, Heidelberg, Germany, was ranked the second most productive author and the first on the most co-cited list, placing him among the top ten in both lists. Another author ranked highly on both lists was Gillies MC (Sydney Eye Hospital, Sydney, Australia).

**TABLE 3:** Top 10 authors and co-cited authors.

No.	Author	Country	Count (%)	Co-cited author	Country	Citation count
1	Bandello F	Italy	77 (2.367%)	Jonas JB	Germany	902
2	Jonas JB	Germany	57 (1.752%)	Haller JA	United States	597
3	Loewenstein A	Israel	49 (1.506%)	Campochiaro PA	United States	542
4	Scott IU	United States	38 (1.168%)	Gillies MC	Australia	516
5	Whitcup SM	United States	34 (1.045%)	Ip MS	United States	487
6	Gillies MC	Australia	33 (1.014%)	Boyer DS	United States	476
7	Kodjikian L	France	33 (1.014%)	Martidis A	United States	395
8	Kuppermann BD	United States	31 (0.953%)	Massin P	France	377
9	Kreissig I	Germany	29 (0.891%)	Klein R	United States	370
10	Sakamoto T	Japan	29 (0.891%)	Brown DM	United States	337

### 3.5 Analysis of Co-Occurring Keywords and Burst Term

**FIGURE 3:**
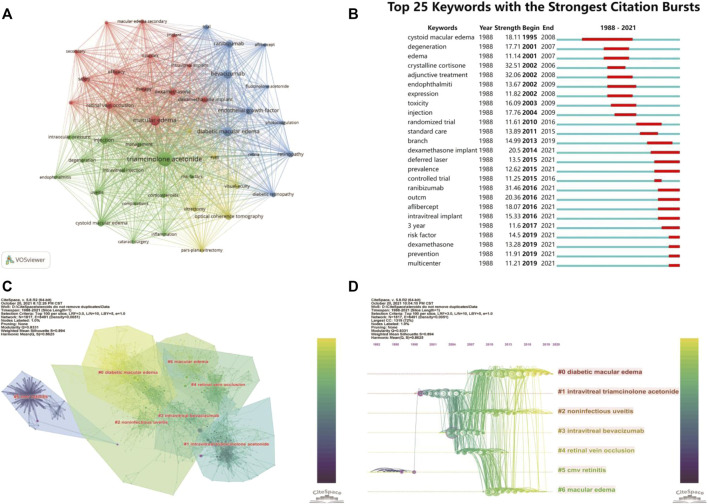
The main keywords and co-citation clusters. **(A)** VOSviewer visualization map of co-occurrence keywords. **(B)** Time trends of burst keywords. **(C)** CiteSpace visualization clusters of co-cited references. **(D)** Timeline view of clusters of co-cited references.

Keywords analysis can reveal hotspots in a topic of study. We merged some different versions of the same terms when we analyzed the keywords, including synonyms (e.g., “triamcinolone acetonide” and “triamcinolone”), different spelling versions (e.g., “triamcinolone acetonide” and “triamcinoloneacetonide”), and abbreviated terms (e.g., “DME” and “diabetic macular edema”). Table 4 shows the top 30 keywords, with most of them falling into two categories. Macular edema and disorders associated with macular edema, such as diabetic macular edema (DME), cystoid macular edema (CME), and RVO, are covered in the disease category. The other group is macular edema therapy, including keywords like TA, injections, and bevacizumab. The rest of the top 30 keywords are related to the efficacy and safety of these treatments. Interestingly, half of the top 6 keywords are VEGF-related terms (Bevacizumab, Endothelial growth factor, and Ranibizumab) (Table 4), suggesting anti-VEGF therapy and steroids therapy in macular edema were often connected. In Figure 3A, we used VOSviewer software to show the primary keywords more compact and aesthetically pleasing.

**TABLE 4:** Top 30 keywords.

No.	Keywords	Count
1	Triamcinolone acetonide	1284
2	Injection	526
3	Bevacizumab	495
4	Macular edema	444
5	Endothelial growth factor	425
6	Ranibizumab	331
7	Retinopathy	317
8	Diabetic macular edema	272
9	Efficacy	265
10	Cystoid macular edema	262
11	Retinal vein occlusion	243
12	Optical coherence tomography	235
13	Safety	227
14	Therapy	197
15	Trial	195
16	Eye	185
17	Visual acuity	167
18	Drug delivery system	167
19	Intraocular pressure	165
20	Management	163
21	Degeneration	146
22	Dexamethasone implant	146
23	Dexamethasone	135
24	Risk factor	125
25	Photocoagulation	117
26	Secondary	116
27	Macular edema secondary	110
28	Pharmacokinetics	106
29	Pars plana vitrectomy	106
30	Outcome	99

We also used CiteSpace’s burst detection function to discover the top 25 terms with the most citation bursts (Figure 3B). A blue line displays the time interval, whereas a red line segment depicts the burst period time of keywords. Similarly, most of the top 25 burst keywords may be classified as disorders connected to macular edema and its treatment. Keywords associated with macular edema, such as cystoid macular edema, degeneration, and edema, began to burst during the early stages. Following that, keywords like crystalline cortisone and adjunctive treatment began to explode, showing that the study focus had shifted to finding solutions to these perplexing issues. Also worth noting, intravitreal implant and VEGF-related terms only had bursts recently (Figure 3B).

### 3.6 Co-cited References and Burst References

#### 3.6.1 Top-Cited References

The most distinctive feature of CiteSpace is co-cited references analysis, resulting in a broad perspective of a scientific subject by selecting the top 100 most cited publications published over 1 year. We compiled a list of the top 12 co-cited references from 1988 to 2021 (Table 5). These references were cited more than 100 times, with the top two receiving more than 200 citations each. The most frequently cited reference was a report on clinical trials (registered with the identifiers NCT00168337 and NCT00168389 at ClinicalTrials.gov), reporting the safety and efficacy of dexamethasone intravitreal implant (Ozurdex, DEX implant) 0.7 and 0.35 mg in the treatment of DME (Boyer et al., 2014). The second most cited paper also reported the results of the intravitreal triamcinolone acetonide (IVTA) clinical trial for treating refractory DME that did not respond well to photocoagulation (Martidis et al., 2002).

**TABLE 5:** Top 12 co-cited references.

No.	Citation count	Author	Reference title	Journal	Year
1	272	Boyer DS	Three-year, randomized, sham-controlled trial of dexamethasone intravitreal implant in patients with diabetic macular edema.	Ophthalmology	2014
2	200	Martidis A	Intravitreal triamcinolone for refractory diabetic macular edema.	Ophthalmology	2002
3	177	Jonas JB	Intravitreal injection of triamcinolone for diffuse diabetic macular edema.	Arch Ophthalmol-Chic	2003
4	153	Haller JA	Randomized, sham-controlled trial of dexamethasone intravitreal implant in patients with macular edema due to retinal vein occlusion.	Ophthalmology	2010
5	149	Haller JA	Dexamethasone intravitreal implant in patients with macular edema related to branch or central retinal vein occlusion.	Ophthalmology	2011
6	141	Massin P	Intravitreal triamcinolone acetonide for diabetic diffuse macular edema - Preliminary results of a prospective controlled trial.	Ophthalmology	2004
7	132	Wells JA	Aflibercept, bevacizumab, or ranibizumab for diabetic macular edema.	New Engl J Med	2015
8	119	Greenberg PB	Intravitreal triamcinolone acetonide for macular oedema due to central retinal vein occlusion.	Brit J Ophthalmol	2002
9	115	Antcliff RJ	Intravitreal triamcinolone for uveitic cystoid macular edema: An optical coherence tomography study.	Ophthalmology	2001
10	111	Jonas JB	Intraocular pressure after intravitreal injection of triamcinolone acetonide.	Brit J Ophthalmol	2003
11	107	Lowder C	Dexamethasone intravitreal implant for noninfectious intermediate or posterior uveitis.	Arch Ophthalmol-Chic	2011
12	102	Campochiaro PA	Sustained delivery fluocinolone acetonide vitreous inserts provide benefit for at least 3 years in patients with diabetic macular edema.	Ophthalmology	2012

#### 3.6.2 Seven Clusters of Co-citation Network

CiteSpace could potentially partition the co-citation network into clusters, displaying firmly related references in one cluster and loosely connected references in another. Words from the titles of the citing articles inside the cluster were used to designate each cluster. The top seven clusters were shown in Figure 3C, including #0 diabetic macular edema, #1 intravitreal triamcinolone acetonide, #2 noninfectious uveitis, #3 intravitreal bevacizumab, #4 retinal vein occlusion, #5 cmv retinitis, and #6 macular edema. These included four clusters about the macular edema-related diseases (clusters #0, 2, 4, and 6), one cluster about IVTA, one about anti-VEGF, and one about complications related to steroids treatment (#5 CMV retinitis).

#### 3.6.3 Timeline Cluster Map

The cluster map could be converted into a timeline format, using the cluster number as the *y*-axis (Figure 3D). The timeline diagram depicted the progression of research in the general field and its seven sub-fields over time. Notably, cluster #5 CMV retinitis was mainly in the early time, and never overlapped with other clusters (Figure 3D); suggesting CMV retinitis is not a major complication of steroids in macular edema treatment. Cluster #0 (DME), #2 (noninfectious uveitis), and #6 (ME, actually mainly about RVO) persisted to recent time, indicating these three ocular diseases are primary indications for steroids therapy of macular edema now (Figure 3D)

#### 3.6.4 High Betweenness Centrality Papers

Some nodes in the timeline-cluster map had purple rings around them, indicating that they had high “betweenness centrality” ratings, which is defined as the number of times a node lies on the shortest path between all pairs of nodes (Abbasi et al., 2011). The top five papers with higher “betweenness centrality” are shown in Table 6. These papers may suggest some emerging trends in this field. Interestingly, two of them published 20 years ago belong to cluster #5 (CMV retinitis). The other three papers are all about IVTA or its complication (glaucoma), suggesting prevention of ocular complications of steroids is still essential now.

**TABLE 6:** Cited papers with the highest “betweenness centrality” among the top 7 clusters.

Rank	Centrality	References	Cluster #
1	0.41	Wingate and Beaumont. (1999): Intravitreal triamcinolone and elevated intraocular pressure.	1
2	0.41	Rothova. (1996): Causes and frequency of blindness in patients with intraocular inflammatory disease.	5
3	0.39	Challa et al. (1998): Exudative macular degeneration and intravitreal triamcinolone: 18 months follow up.	5
4	0.16	Jonas et al. (2003b): Intraocular pressure after intravitreal injection of triamcinolone acetonide.	1
5	0.1	Massin. (2004): Intravitreal triamcinolone acetonide for diabetic diffuse macular edema: preliminary results of a prospective controlled trial.	3

#### 3.6.5 Details of Cluster #1 (IVTA)

In cluster #1 (IVTA), Jonas JB was the author of half of the top six co-cited references (Table 7), including a case report and two clinical trial articles (Jonas et al., 2003a; Jonas et al., 2003b; Jonas and Söfker, 2001). He was also the author of the top five citing articles, citing most publications in this field (coverage count) (Table 7). All these five citing papers were reviews, and two were in German (Jonas et al., 2003c; Jonas et al., 2004; Jonas et al., 2005a; Jonas, 2005; Jonas, 2006). As previously stated, Jonas JB ranked first among the most cited authors, and we now know that he made a significant contribution to the treatment of DME using IVTA. The clinical article by Martidis et al. published in 2002 was the most prominent in cluster #1 (IVTA). Unlike Jonas JB’s clinical trial papers, all of the patients involved in this investigation had received at least two prior sessions of laser photocoagulation yet still showed residual macular edema (Martidis et al., 2002).

**TABLE 7:** Cited references and citing articles of Cluster #1 intravitreal triamcinolone acetonide.

Cluster #1 intravitreal triamcinolone acetonide			
Cited references		Citing articles	
Citation count	Author (Year) Journal	Coverage	Author (Year) title
	Volume, Page	Count	
200	Martidis et al. (2002) Ophthalmology, 109, 920	108	Jonas. (2006) Intravitreal triamcinolone acetonide: a change in a paradigm.
177	Jonas et al., 2003a Arch Ophthalmol-Chic, 121, 57	91	Jonas. (2005) Intravitreal triamcinolone acetonide for treatment of intraocular oedematous and neovascular diseases.
119	Greenberg et al. (2002) Brit J Ophthalmol, 86, 247	88	Jonas. (2005) Intravitreal triamcinolone acetonide for treatment of intraocular proliferative, exudative, and neovascular diseases.
115	Antcliff. (2001) Ophthalmology, 108, 765	60	Jonas et al. (2004) Intravitreal triamcinolone acetonide for the treatment of intraocular edematous and neovascular diseases.
111	Jonas et al. (2003b) Brit J Ophthalmol, 87, 24	45	Jonas et al., 2003c Treatment of oedematous, proliferative and neovascular diseases by intravitreal triamcinolone acetonide.
97	Jonas and Söfker. (2001) Am J Ophthalmol, 132, 425	45	Thompson, (2006) Cataract formation and other complications of intravitreal triamcinolone for macular edema.

#### 3.6.6 Details of Cluster #0 (DME), Cluster #2 (Noninfectious Uveitis), Cluster #4 (RVO) and Cluster #6 (Macular Edema)

Macular edema can be caused by a variety of ocular diseases, including DME, uveitis, and RVO (Cunningham et al., 2008). In cluster #0 (DME) (Table 8), the paper about dexamethasone intravitreal implant (Boyer et al., 2014) was the top-cited reference, and it was also the top co-cited reference among all clusters (Table 5). In cluster #2 (noninfectious uveitis), the paper about dexamethasone intravitreal implant for uveitis was the most cited reference (Lowder et al., 2011), which indicated that DEX implant was safe and effective for the treatment of intermediate and posterior uveitis (Table 9). By employing the DEX implant, they were able to make a significant contribution to the treatment of uveitic macular edema. Regardless of the citing articles or references, all of the publications in cluster #4 (RVO) and cluster #6 (macular edema) were about the treatment of macular edema caused by RVO (Table 10 and Table 11). These two clusters of co-cited references were all clinical trials or case reports examining the efficacy and safety of medications to treat RVO-induced macular edema. Apart from IVTA, two other therapies were used: DEX implant and ranibizumab. DEX implant is a slow-releasing biodegradable implant indicated for injection every 6 months and is a combination of medication and drug delivery mechanisms (Gaballa et al., 2021). Ranibizumab is a recombinant monoclonal antibody that can block VEGF-A (Rosenfeld et al., 2006).

**TABLE 8:** Cited references and citing articles of Cluster #0 diabetic macular edema.

Cluster #0 diabetic macular edema			
Cited references		Citing articles	
Citation count	Author (Year) Journal	Coverage	Author (Year) title
	Volume, Page	Count	
272	Boyer. (2014) Ophthalmology, 121,1904	40	Cicinelli, Maria Vittoria (2020) The current role of steroids in diabetic macular edema.
132	Wells et al. (2015) New Engl J Med, 372, 1193	36	Bandello, Francesco (2013) Pathophysiology and treatment of diabetic retinopathy.
102	Campochiaro et al. (2012) Ophthalmology, 119, 2125	31	Bandello et al. (2014) Pharmacological approach to diabetic macular edema.
98	Nguyen et al. (2012) Ophthalmology, 119, 789	31	Cebeci, Zafer (2015) Role of implants in the treatment of diabetic macular edema: focus on the dexamethasone intravitreal implant.
92	Chang-Lin et al. (2011) Invest Ophth Vis Sci, 52, 80	29	Urbancic, Mojca (2019) Dexamethasone implant in the management of diabetic macular edema from clinician’s perspective.
91	Boyer et al. (2011) Retina-J Ret Vit Dis, 31, 915	29	Lambiase et al. (2014) An update on intravitreal implants in use for eye disorders.

**TABLE 9:** Cited references and citing articles of Cluster #2 noninfectious uveitis.

Cluster #2 noninfectious uveitis			
Cited references		Citing articles	
Citation count	Author (Year) Journal	Coverage	Author (Year) title
	Volume, Page	Count	
107	Lowder et al. (2011) Arch Ophthalmol-Chic, 129, 545	24	Heiligenhaus et al. (2014) Statement of the german ophthalmological society, of the retinological society and the professional association of german oculist for intravitreal therapy for makulaodems in uveitis.
58	Kuppermann et al. (2007) Arch Ophthalmol-Chic, 125, 309	24	Heiligenhaus et al. (2014) Statement of the german ophthalmological society, the retina society and the professional association of eye doctors in germany for intravitreal treatment of macular edema in uveitis.
50	Kiddee et al. (2013) Surv Ophthalmol, 58, 291	19	Lambiase et al. (2014) An update on intravitreal implants in use for eye disorders.
40	Tomkins-Netzer et al. (2014) Ophthalmology, 121, 1649	19	Ossewaarde-van Norel, Annette (2011) Clinical review: update on treatment of inflammatory macular edema.
36	Khurana et al. (2014) Ophthalmology, 121, 67	18	Robinson, Michael R (2012) Pharmacologic and clinical profile of dexamethasone intravitreal implant.

**TABLE 10:** Cited references and citing articles of Cluster #4 retinal vein occlusion.

Cluster #4 retinal vein occlusion			
Cited references		Citing articles	
Citation count	Author (Year) Journal	Coverage	Author (Year) title
	Volume, Page	Count	
92	Ip et al. (2009) Arch Ophthalmol-Chic, 127, 1101	41	Feltgen et al. (2010) Intravitreal drug therapy for retinal vein occlusion - pathophysiological mechanisms and routinely used drugs.
88	Scott et al. (2009) Arch Ophthalmol-Chic, 127, 1115	33	Macdonald, Derek (2014) The abcs of rvo: a review of retinal venous occlusion.
57	Iturralde et al. (2006) Retina-J Ret Vit Dis, 26, 279	33	Chatziralli, Irini P (2014) Branch retinal vein occlusion: treatment modalities: an update of the literature.
43	Jonas. (2005) Eye, 19, 65	26	Siegel, Ruth Axer (2012) Intravitreal bevacizumab treatment for macular edema due to branch retinal vein occlusion in a clinical setting.
34	Rosenfeld et al. (2005) Ophthal Surg Las Im, 36, 336	21	Sarao et al. (2014) Pharmacotherapy for treatment of retinal vein occlusion.
33	Rabena et al. (2007) Retina-J Ret Vit Dis, 27, 419	21	Braithwaite, T (2010) Anti-vascular endothelial growth factor for macular edema secondary to central retinal vein occlusion.
29	Prager et al. (2009) Brit J Ophthalmol, 93, 452	21	Shahsuvaryan, Marianne L (2012) Therapeutic potential of intravitreal pharmacotherapy in retinal vein occlusion.

**TABLE 11:** Cited references and citing articles of Cluster #6 macular edema.

Cluster #6 macular edema			
Cited references		Citing articles	
Citation count	Author (Year) Journal	Coverage	Author (Year) title
	Volume, Page	Count	
153	Haller et al. (2010) Ophthalmology, 117, 1134	27	Macdonald, Derek (2014) The abcs of rvo: a review of retinal venous occlusion.
149	Haller et al. (2011) Ophthalmology, 118, 2453	20	Wang, Jia-Kang (2016) A review of randomized trials of approved pharmaceutical agents for macular edema secondary to retinal vein occlusion.
70	Brown et al. (2011) Ophthalmology, 117, 1124^a^	17	Garweg, Justus G (2016) Retinal vein occlusion and the use of a dexamethasone intravitreal implant (ozurdexa (r)) in its treatment.
65	Campochiaro et al. (2010) Ophthalmology, 117, 1102	17	Ramezani, Alireza (2014) Three intravitreal bevacizumab versus two intravitreal triamcinolone injections in recent onset central retinal vein occlusion.
49	Campochiaro et al. (2011) Ophthalmology, 118, 2041	17	Sarao et al. (2014) Pharmacotherapy for treatment of retinal vein occlusion.
48	Capone et al. (2014) Retina-J Ret Vit Dis, 34, 342	17	Coscas, Gabriel (2014) Retreatment with ozurdex for macular edema secondary to retinal vein occlusion.
47	Brown et al. (2011) Ophthalmology, 118, 1594	17	Maggioa, Emilia (2014) Intravitreal dexamethasone implant for macular edema secondary to retinal vein occlusion: 12-month follow-up and prognostic factors.

a Here, we need to mention that CiteSpace could only analyze the publications in the WOS core collection database, so we only downloaded these publications. However, the references cited by these articles may not be collected in this database, so information on such references would have some discrepancies. In this cluster, the third cited reference was not the one listed in the results of CiteSpace, and it was only collected by the Medline database rather than the WOS Core Collection database. Actually, the primary one given by CiteSpace was a letter by the same author team to reply to some questions about the treatment of age-related macular degeneration by using ranibizumab, which was obviously irrelative with our research topic. Additionally, CiteSpace also gave a DOI number that did not match this article, but the number helped us to find out the matched and right article shown in Table 9 with an asterisk. Because this article was published by the same team as the fourth, fifth, and seventh articles in Table 9, and they were all reports of the efficacy and safety results of the clinical trials about the treatment of RVO-induced macular edema by using ranibizumab, so we used this article to replace the former one instead of straight deleting it in Table 9.

## 4 Discussion

The first article on this research topic was published in 1988, which described the successful use of steroids drops and sub-Tenon injection to treat cystoid macular edema (CME) following cataract extraction (Suckling and Maslin, 1988), suggesting that local steroids are effective in the treatment of pseudophakic CME. However, only a few studies were published over the next decade, mainly about systemic steroids on uveitis-related macular edema (Nussenblatt et al., 1991), steroid-induced ocular complications in the treatment of aphakic or pseudophakic CME (Melberg and Olk, 1993), and steroids sub-Tenon injections in uveitis patients with cystoid macular edema (Yoshikawa et al., 1995). CME was the top keyword during that time (Figure 3B). Steroid research in macular edema was still in its infancy at that time.

The first research wave lasting for one decade began from 2003 (Figure 2A), shortly after the first reports of human intravitreal injections of crystalline cortisone in the treatment of DME (Jonas and Söfker, 2001), IVTA for uveitic cystoid macular edema (Antcliff et al., 2001), IVTA for macular edema due to CRVO (Greenberg et al., 2002) and refractory DME (Martidis et al., 2002). These four papers are pioneering works, coming from Europe (Jonas and Söfker, 2001; Antcliff et al., 2001) and the United States (Martidis et al., 2002; Greenberg et al., 2002), and are all top-cited in this field (Table 5). At this time, the average publication per year on this topic was more than 110 papers. The second research wave lasting almost another decade began from 2013 to 2014 (Figure 2A), the average publication per year on this topic was more than 220 papers. This wave of research may be prompted by good results of the MEAD study demonstrating the efficacy of dexamethasone implant in the treatment of DME (Boyer et al., 2014), and the FDA-approved use of sustained-release biodegradable dexamethasone (Ozurdex) for the treatment of DME in 2014. The paper of the MEAD trial is the top-cited reference among all publications in this field (Boyer et al., 2014).

From the perspective of countries and authors, the United States was the leading contributor in this field with 913 publications, followed by Germany and Italy (Table 1). The top three countries were all developed countries in Europe and America. The research output from these countries may be associated with major pioneer researchers in this field and substantial financial support. Indeed, most of the top authors and co-cited authors were from Europe and America (Table 3). Europe and American researchers initiated both waves of research in this field. Among the top 10 countries, there are four countries from Asia (Japan, South Korea, India, and China), likely due to the vast number of patients in Asia. Sakamoto T was the only top-productive author from these countries; however, no top-cited authors came from these countries (Table 3).

Bandello F published the most papers. His significant contribution to this field is three clinical trials regarding the use of DEX intravitreal implant in macular edema caused by RVO (Haller et al., 2010; Haller et al., 2011) and DME (Boyer et al., 2014). Following Bandello F, Jonas JB, Loewenstein A, Scott IU, Whitcup SM and Gillies MC were the top six most productive authors with cooperative relationships. Jonas JB and Gillies MC were also top-cited authors (Table 3). Jonas JB is a comprehensive ophthalmologist and Chairman of the Department of Ophthalmology of the Medical Faculty Mannheim of Heidelberg University. His major contribution to this field is the first report on intravitreal injections of crystalline cortisone in the treatment of DME patients (Jonas and Söfker, 2001), IVTA for DME (Jonas et al., 2003a; Jonas et al., 2006), and IVTA-induced intraocular pressure (Jonas et al., 2003b; Jonas et al., 2005b). Gillies MC is a Professor at Save Sight Institute, University of Sydney. His major contribution to this field is similar to Bandello F, including the use of DEX intravitreal implant in macular edema caused by RVO (Haller et al., 2010; Haller et al., 2011) and DME (Sutter et al., 2004; Gillies et al., 2006). These researchers are considered world leaders in this critical research field and their studies will continue to influence the future development of steroids in the treatment of macular edema.

From the perspective of journals and co-cited journals, *IOVS* published the most papers, followed by *Retina, EJO, and AJO*. The average number of citations for each document is up to 20 times, which fully indicates that researchers obtained international recognition in this field. Co-citation analysis revealed that *Ophthalmology* had been the most co-cited journal (Table 2), with the highest impact factor. Indeed, more than half of the 12 top-cited references in this field were published in *Ophthalmology* (Table 5), all about clinical trials regarding DEX implants (Haller et al., 2010; Haller et al., 2011; Boyer et al., 2014), IVTA (Antcliff et al., 2001; Martidis et al., 2002; Massin et al., 2004), and FA inserts (Campochiaro et al., 2012). These studies significantly promote the safety and efficacy of steroids use in treating of macular edema.

Keywords can provide immediate information about hotspots in the field. Word analysis can show how the research concepts evolve during a period. In our study, keywords analysis showed that the high-frequency keywords belong to the disease category (including DME, RVO) and therapy category (including TA, DEX implant, and VEGF-related terms). Interestingly, intravitreal implant and VEGF-related terms were among these top keywords and had bursts recently (Figure 3B). These keywords captured this field’s current and future direction (Sacconi et al., 2019). Anti-VEGF agents, including bevacizumab, ranibizumab, and aflibercept, are the first-line treatment for DME (Brown et al., 2013; Wells et al., 2015) and macular edema secondary to RVO (Boyer et al., 2012; Holz et al., 2013; Schmidt-Erfurth et al., 2019). While they are more effective and have fewer side effects than steroids (Gao et al., 2019), a significant proportion of patients do not respond well and repeated intravitreal injections are required to maintain the visual benefits (Engman et al., 2011). Steroids generally only have short-term effects, but new DEX and FA sustained-release devices have been developed, such as Ozurdex (DEX implant), Iluvien (FA implant), and YUTIQ (FA implant) (Testi and Pavesio, 2019; McGregor et al., 2021), which offer a longer duration of action and can reduce the number of intravitreal injections required (DEX for 4–6 months; FA for 3 years) (Bandello et al., 2014). These steroids implants or inserts are commonly used as a second-line treatment for DME patients without significant response to anti-VEGF therapies (Tan et al., 2017; He et al., 2018; Fallico et al., 2021), but are also considered as valid therapeutic options in the treatment of RVO-induced macular edema because of their anti-inflammatory, anti-angiogenic, and anti-edema properties (Haller et al., 2010; Haller et al., 2011; Castro-Navarro et al., 2021). Although some patients require repeated steroid injections, the long duration between treatments is still a considerable advantage of these drugs (Scaramuzzi et al., 2015).

Betweenness centrality results from cluster analysis of co-citation network identified important papers about complications of ocular steroids treatment (Table 6). Although CMV retinitis was mainly reported almost 20 years ago based on the timeline-cluster map (Figure 3D), it was frequently reported recently in patients receiving intravitreal injection of steroids (such as IVTA) or steroids implants (Jaissle et al., 2004; Saidel et al., 2005; Ufret-Vincenty et al., 2007; Witmer and Connolly, 2021). Increased intraocular pressure (IOP) and cataract formation are common side effects of various types of steroids (Jonas et al., 2005b; Yang et al., 2015). According to a Cochrane systematic review, cataracts progressed in 5 or 6 out of 10 patients treated with steroids, and IOP increased in roughly 3 out of 10 patients, which is significantly greater than the control group (Rittiphairoj et al., 2020). These possible side effects must be carefully monitored when using steroids to treat macular edema.

By systematically combining the publications during the past three decades, we show the dynamic development process, hotspots, research trends, and structural relationship of different aspects of steroids treatment of macular edema. There are some limits to this study. First, the analysis of this study is only based on publications in the WOS core collection database. Although this collection includes most of the research publications in this field, other databases may provide broader coverage, such as PubMed and Scopus. Second, the research results of this paper are completed based on CiteSpace and VOSviewer, and the machine algorithm may not be perfect and can induce some bias. For instance, IVTA is a major cluster in the keyword and cluster analysis (Figures 3C,D), DEX and FA, on the other hand, are only found on the periphery of the term visualization map and do not even make the top ten in cluster analysis. However, the number of IVTA publications has decreased dramatically, while DEX and FA implants have received increasing attention in recent clinical trials (Castro-Navarro et al., 2021; Fallico et al., 2021). This trend has not been captured in our study.

Combined therapy with anti-VEGF and suprachoroidal corticosteroids may overcome the limits of both agents and increase the therapeutic efficacy (Sacconi et al., 2019). Our study also misses this trend, as many new clinical trials on these strategies are currently being conducted, and results are not published yet. More outcomes from these trials will help us build a better approach for treating macular edema in the future after balancing the benefits and limits of various therapeutic strategies and the patient’s medical needs.

## 5 Conclusion

We are at the second research wave of using steroids to treat macular edema with more than 200 publications every year. The United States and Europe led in this field by contributing the most important papers. Papers published in a specialty journal such as *Ophthalmology* will attract more attention than papers published in comprehensive journals. While anti-VEGF therapy is the first-line treatment for DME and RVO-induced macular edema, steroids implant is a valid option for these DME patients not responding to anti-VEGF therapy and non-DME patients with macular edema. Combined therapy with anti-VEGF and steroids agents is essential for future research.

## Data Availability

The raw data supporting the conclusions of this article will be made available by the authors, without undue reservation.
